# Genetic characterization of wild-type measles viruses isolated in China, 2006-2007

**DOI:** 10.1186/1743-422X-7-105

**Published:** 2010-05-25

**Authors:** Yixin Ji, Songtao Xu, Yan Zhang, Zhen Zhu, Naiying Mao, Xiaohong Jiang, Chao Ma, Peishan Lu, Changyin Wang, Yong Liang, Huanying Zheng, Yang Liu, Defang Dai, Lei Zheng, Jianhui Zhou, Shuang Wang, Zhenying Zhang, Shengwei Wu, Lijuan Nan, Li Li, Xiaofeng Liang, David Alexander Featherstone, Paul A Rota, William J Bellini, Wenbo Xu

**Affiliations:** 1 WHO WPRO Regional Reference Measles Lab and State Key Laboratory for Molecular Virology & Genetic Engineering, National Institute for Viral Disease Control and Prevention, China Center for Disease Control and Prevention, Beijing 100050, China; 2 National Immunization Program, China Center for Disease Control and Prevention, China; 3 Jiangsu Provincial Center for Disease Control and Prevention, China; 4 Shandong Provincial Center for Disease Control and Prevention, China; 5 Hebei Provincial Center for Disease Control and Prevention, China; 6 Guangdong Provincial Center for Disease Control and Prevention, China; 7 Tianjin Provincial Center for Disease Control and Prevention, China; 8 Hunan Provincial Center for Disease Control and Prevention, China; 9 Shanxi Provincial Center for Disease Control and Prevention, China; 10 Jilin Provincial Center for Disease Control and Prevention, China; 11 Henan Provincial Center for Disease Control and Prevention, China; 12 Guizhou Provincial Center for Disease Control and Prevention, China; 13 Neimeng Provincial Center for Disease Control and Prevention, China; 14 Immunization, Vaccines and Biologicals, World Health Organization, Geneva, Switzerland; 15 Division of Viral Diseases, Centers for Disease Control and Prevention, 1600 Clifton Road, Atlanta, GA 30333, USA

## Abstract

Molecular characterization of wild-type measles viruses in China during 1995-2004 demonstrated that genotype H1 was endemic and widely distributed throughout the country. H1-associated cases and outbreaks caused a resurgence of measles beginning in 2005. A total of 210,094 measles cases and 101 deaths were reported by National Notifiable Diseases Reporting System (NNDRS) and Chinese Measles Laboratory Network (LabNet) from 2006 to 2007, and the incidences of measles were 6.8/100,000 population and 7.2/100,000 population in 2006 and 2007, respectively. Five hundred and sixty-five wild-type measles viruses were isolated from 24 of 31 provinces in mainland China during 2006 and 2007, and all of the wild type virus isolates belonged to cluster 1 of genotype H1. These results indicated that H1-cluster 1 viruses were the predominant viruses circulating in China from 2006 to 2007. This study contributes to previous efforts to generate critical baseline data about circulating wild-type measles viruses in China that will allow molecular epidemiologic studies to help measure the progress made toward China's goal of measles elimination by 2012.

## Introduction

Measles virus (MeV) is a single-stranded, negative-sense RNA virus, belonging to the genus *Morbillivirus*, family *Paramyxoviridae*, order *Mononegavirale*s. Measles is a vaccine-preventable disease, but is still a major killer of infants worldwide. During 2000-2008, global measles mortality declined by 78%, from an estimated 733,000 deaths in 2000 to 164,000 in 2008 [1,2]. Measles continues to be a leading cause of childhood morbidity and mortality in developing countries and an outbreak threat in the majority of countries, despite the availability of an effective vaccine for over 40 years [3]. Molecular epidemiologic studies can help to measure transmission pathways and to clarify epidemiological links during outbreaks. MeV is considered serologically monotypic although genetic heterogeneity has been detected among wild type strains [1,4]. Twenty-three genotypes (A, B1-B3, C1-C2, D1-D10, E, F, G1-G3 and H1-H2) have been recognized by World Health Organization (WHO); however five genotypes (B1, D1, E, F, and G1) are considered inactive since they have not been detected in the past 15 years [5,6]. Measles surveillance can also help to measure the success of measles vaccination programs by documenting the interruption of transmission of the endemic viral genotype(s) [7,8].

The WHO Regional Committee of the Western Pacific Region (WPR) formally declared a measles elimination goal in 2005, and established a target date of 2012 for regional measles elimination [9,10]. In China, a laboratory surveillance network for measles was established from 2001 to provide serologic confirmation of measles infection by IgM testing and to support measles surveillance. The measles laboratory network has been established in all 31 provinces and includes 331 prefecture-level laboratories which perform most of the serological testing for case confirmation. The National Measles Laboratory (NML) has played an important role in measles control and measles virus surveillance and was appointed as a regional reference lab in WPR in 2003. To reach the goal of measles elimination by 2012 in China, the Ministry of Health (MOH) proposed a comprehensive vaccination and surveillance programme in 2006 (Measles Elimination Action Plan from 2006-2012 issued by MOH). "Measles elimination action plan" keeps high routine coverage of the first dose at 8 month and the second dose at 18-24 months and combines with supplemental immunization activities among the age from 8 month to 14 years old.

This study describes the genetic characterization of wild-type measles viruses reported from 24 of 31 provinces in China from 2006-2007. Five hundred and sixty-five wild-type measles viruses were isolated from throat swab or urine specimens collected from outbreak and sporadic cases. The results documented the continued circulation of genotype H1 viruses throughout China.

## Results

### Epidemiology

**Figure 1:**
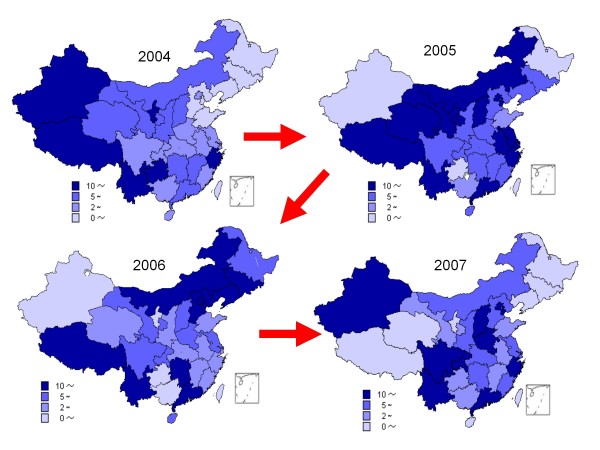
**Measles Incidence in China 2004-2007**. The maps show incidence of measles by province for the years 2004, 2005, 2006 and 2007. Color code at bottom of each map indicates the incidence rate. In 2004, Xinjiang, Xizang, Yunnan, Guizhou, Gansu and Zhejiang: measles incidence was >10/100,000. In 2005, Jiangsu, Shanghai, Beijing, Tianjin, Zhejiang, Fujian, Anhui, Guangdong, Inner Mongolia, Shanxi, Gansu, Qinghai, Xizang and Yunnan: the incidence of measles was >10/100,000

In 2006 and 2007, the incidence of measles was 6.8/100,000 and 7.2/100,000, respectively, with a total of 100,267 and 109,827 cases and 35 and 66 fatalities reported by NNDRS. In 2004, measles incidence was > 10/100,000 in 6 of 31 provinces (Xinjiang, Xizang, Yunnan, Guizhou, Gansu and Zhejiang) which were located in the developing areas of China with the exception of Zhejiang province. However, in 2005, the incidence of measles was > 10/100,000 in 14 of 31 provinces, which included 8 developed (Jiangsu, Shanghai, Beijing, Tianjin, Zhejiang, Fujian, Anhui and Guangdong) and 6 developing (Inner Mongolia, Shanxi, Gansu, Qinghai, Xizang and Yunnan) provinces (Figure 1). Though the 14 provinces which had measles incidence >10/100,000 population in 2005 decreased to 8 and 9 provinces in 2006 and 2007, respectively, measles virus of the H1 genotype was still detected in all 24 provinces sampled and was associated with over 1500 measles outbreaks throughout China. From 2005 to 2007, measles incidence increased significantly in those developed provinces which previously had low measles incidence rates (Figure 1).

**Figure 2:**
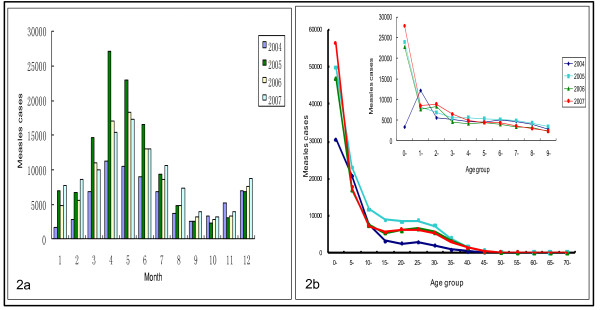
**Age-specific and Month-specific of cases of measles in China, 2004-2007**. Figure 2a shows month-specific of measles cases from 2004-2007 with different color bar. Figure 2b shows age-specific of measles from 0-70 years old and 0-10 years old.

In 2006 and 2007, measles cases in those < 1 and ≥15 years old also increased compared with 2004 and had another peak at 15 to 35 years (Figure 2b). The age group specific incidence of measles cases reported was still highest among those under 15 years old, especially those under one year of age. Approximately 25% of measles cases occurred in infants 0-1 year of age from 2005 to 2007, compared with 9.0% in 2003 and less than 10% in 2004 (from NNDRS), and the peak appeared between April and May in most years (Figure 2a).

### Molecular characterization

**Figure 3:**
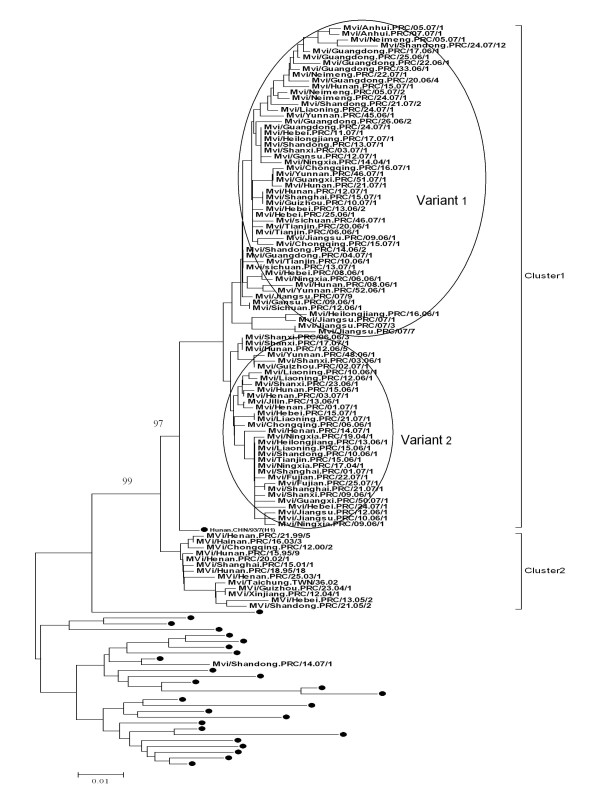
**Phylogenetic tree of measles wild-type virus strains of China in 2006-2007 and WHO reference MeV strains (black dots) based on the 456 nucleotide sequences coding for the COOH-terminus of the nucleoprotein**. The dendrogram was created with MEGA4 software and the neighbor-joining method (500 bootstraps). Genetic distances are represented as numbers of nucleotide differences between strains.

**Figure 4:**
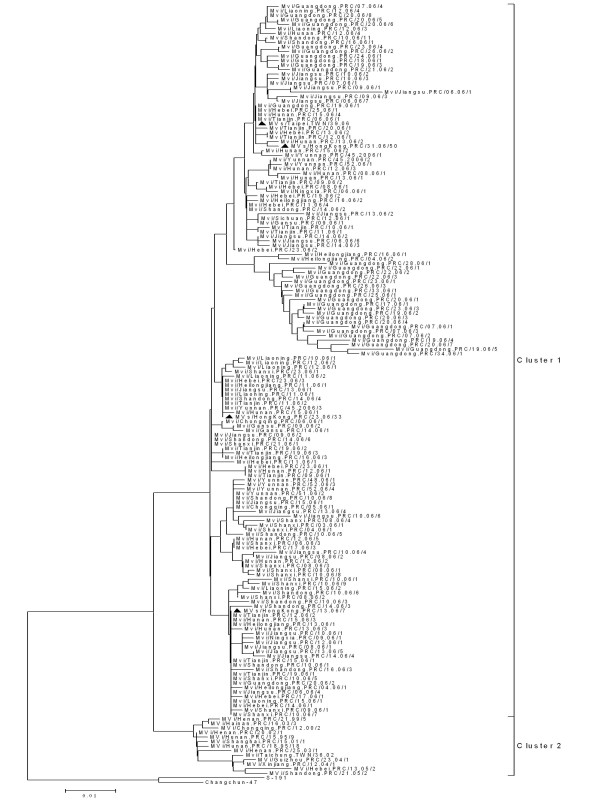
**Phylogenetic tree of measles wild-type virus strains of China in 2006 and H1 reference MeV strains based on the 450 nucleotide sequences coding for the COOH-terminus of the nucleoprotein**. The black triangles indicate MeV isolates Taiwan or Hong Kong China.

**Figure 5:**
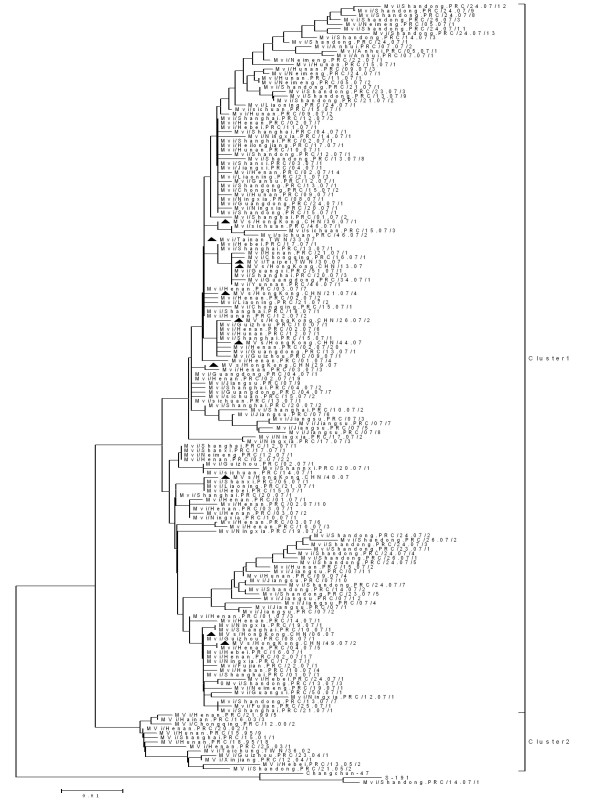
**Phylogenetic tree of measles wild-type virus strains of China in 2007 and H1 reference MeV strains based on the 450 nucleotide sequences coding for the COOH-terminus of the nucleoprotein**. The MeV isolates from Taiwan and Hong Kong China were identified by a BLAST search from of GenBank (shown by black triangles).

In the study, 565 wild-type measles viruses were collected from 24 of 31 provinces in China during the 2006-2007 measles epidemic (Table 1). The swab or urine specimens were collected from outbreak and sporadic cases. Virus isolation was conducted by provincial laboratories and isolates were transported to the NLM in Beijing for molecular characterization. In this study, with the exception of one genotype A strain from Shandong province identified as a vaccine reaction, all strains were members of cluster 1 of genotype H1 and no cluster 2 [11] of H1 genotype were detected in 2006-2007 (Figure 3,4, 5). The results of the phylogenetic analysis from nucleotide positions 1230 to 1685 of the sequences of N gene are shown in Figures 3, 4 and 5. The clustering of MeVs in China within the genotype H1 was supported by a significant bootstrap value (500 replicates) of 97% (Figure 3). It was found that genotype H1 was still the predominant circulating genotype in China during 2006-2007.

**Table 1:** The list of measles virus isolates in 2006-2007.

Province	Number		Genotype	Province	Number		Genotype
	06	07	H1		06	07	H1
Hunan	18	11	cluster1	Jilin	10		cluster1
Henan	1	65	cluster1	Hebei	30	8	cluster1
Shandong	21	61	cluster1, A*	Guangdong	39	18	cluster1
Sichuan	2	12	cluster1	Neimenggu	7	10	cluster1
Jiangsu	45	14	cluster1	Shanghai		24	cluster1
Ningxia	2	19	cluster1	Shanxi	27	5	cluster1
Jiangxi	1	5	cluster1	Yunnan	14	1	cluster1
Shaanxi		2	cluster1	Tianjin	16		cluster1
Guangxi	1	14	cluster1	Liaoning	11	6	cluster1
Heilongjiang	7	1	cluster1	Anhui		3	cluster1
Guizhou		12	cluster1	Chongqing	5	6	cluster1
Gansu	7	1	cluster1	Fujian		3	cluster1
				Total	565		

*Note, one measles virus of genotype A from Shandong provinces was isolated from a vaccine-derived case.

Genotype H1 has the greatest degree of intratypic variation among all of the genotypes, with sequences falling into two major clusters, previously designated as cluster 1 and cluster 2 [12,15]. The sequences of genotype H1 viruses from 2006 to 2007 all were within cluster 1 (Figure 3), and two main branches within this cluster were visible (Figure 3). The mean nucleotide difference between these two branches of variants was 2.2%.

Compared with the WHO reference strains sequences of all genotypes, the range of the nucleotide sequence homologies of the 564 genotype H1 strains was 86.6%-93.5%. With the exception of one vaccine-derived case, all of the 564 measles viruses in 2006-2007 differed from the vaccine strains in China, S191 and Changchun-47, by no more than 10.1% at the nucleotide level and 16.0% at amino acid level in the standard 450 nucleotide region of the N gene that is used for genotyping.

## Discussion

Although the average incidence of measles in China decreased in 2006-2007 compared with 2005, however measles incidence was >10/100,000 in 8 and 9 developed provinces in 2006 and 2007 respectively (Figure 1), reaching the goal of measles elimination by 2012 will be a challenge. Two reasons may account for the recent increase in measles cases in China. Over 30% of measles cases from 2005 to 2007 in China were in the "floating population" (persons without a permanent residence card for the place in which they live) that account for up to 50% of the population in some developed provinces and big cities [16]. The floating population is not able to access routine immunization services outside their official residential area and is likely to constitute a large proportion of the immunity gap in China. The proportion of floating people has increased since 2005 following migration from rural based developing provinces to the more urban based, developed provinces. A high proportion of measles cases in young adults in cities may be responsible for the measles incidence increase among the developed provinces from 2005 to 2007 as this group received only a single dose of measles vaccine as infants.

With the attainment of Universal Childhood Immunization goals [17], measles morbidity in China reached lows in 1995. During 1995-2004, the incidence of measles was approximately 6/100,000 population every year. There was a resurgence of measles cases in 2005 in China where the incidence increased to 10/100,000 population. In 2006, the MOH issued an activity plan to achieve measles elimination by 2012. The plan recommended that the age of the first dose of measles-containing vaccine (MCV1) remain at 8 months, and the age of the second dose of measles-containing vaccine (MCV2) change from 7 years to 18-24 months. China's reported two dose MCV coverage ranged from 84.1% to 96.4% during 2003-2007, with a mean of 92.5% [10]. The incidence dropped to 6.8/100,000 in 2006 and 7.2/100,000 in 2007, because of the measles supplementary immunization activities (SIAs) in some high incidence provinces. In China, SIAs during 2003-2008 reached approximately 101 million children and adolescents in 14 provinces [10]. The age-specific incidence of measles cases was highest among young children and declined with increasing age from 2006-2007. However, the incidence rate had 2 peaks, in infants (< 1 year old), and in adolescents and adults (>15 years old), compared to 1995-2004. Although the developed provinces had different incidence rates, there were no major differences in the age distribution of cases. Furthermore, the change to the measles vaccine schedule, in which the second dose administered to children was changed from 7 year old to 18-24 months in 2005, could have accounted for changing the age distribution.

The virologic surveillance for measles virus that began in China in 1993 is one of the most thorough programs in the global network. Through this time only a single genotype, H1, has been detected in China. Measles surveillance in other countries in Asia has confirmed that endemic circulation of genotype H1 is limited to only China [18]. Analyses conducted during the early part of the last decade identified two lineages within the Chinese H1 viruses and these were designated cluster 1 and cluster 2 with cluster 1 viruses being the most frequently detected strains. The genetic analysis of wild-type measles isolated in 2006 and 2007 indicated that cluster 1 is still the most frequently detected lineage in China. There were no cluster 2 viruses isolated during 2006-2007, compared with 26.9% of all strains detected in 1993-2000 and 5.4% in 2001-2005 [12,13,15,16]. This finding suggested that measles vaccination programme was able to reduce the genetic diversity of circulating measles virus from 1993 to 2007 in China. Even though the genotype H1 viruses in China are genetically diverse, no geographic restriction of particular lineages is evident. The major neutralization sites and N-glycosylation sites in the hemagglutinin were not changed in the more recent genotype H1 viruses compared to those isolates. Compared China vaccine strain, the 719th nucleotide of H1 wild strains in China was changed from G to A in amino acid mutation from Ser to Asn. Furthermore, one N-glycosylation site in 240th amino acids was loosen because of the mutation [14]. Therefore, the amino acid mutation in the H protein of the Chinese viruses did not appear to the result in loss of mayor neutralization epitopes by cross antibody induced following vaccination.

MeVs with identical sequences were associated with outbreaks in multiple provinces, and these lineages were detected in multiple provinces for several years. The sequences from the recent genotype H1 viruses that were imported into Europe, Taiwan and Hong Kong showed 100% sequence identity with genotype H1 viruses that were circulating in mainland China or were associated with international importation of virus [19]. Molecular epidemiologic studies of measles virus are an important component of measles surveillance in China because they will help to document interruption of transmission of the endemic genotype, H1, a key criterion for verification of measles elimination. Measles surveillance is also necessary to monitor the stability of the neutralization epitopes on the viral hemagglutinin.

## Conclusion

This study reports measles surveillance from 24 provinces of China during 2006 to 2007. The results confirmed that genotype H1-cluster 1 is the endemic genotype circulating in China from 2006 to 2007. Genetic analyses of viral samples from the Chinese laboratory network were consistent with endemic measles, in that multiple chains of transmission were evident. However a reduction in genetic diversity suggests that recently introduced enhanced measles control strategies are having an impact in China.

## Materials and methods

### Epidemiologic data

Numbers and descriptive information of measles cases and deaths in this report were obtained from the National Notifiable Diseases Reporting System of China CDC (NNDRS). Population denominators for calculation of incidence and mortality rates were determined on the basis of data reported by the National Bureau of Statistics. Epidemiologic data were analyzed by using Microsoft Excel and Epi Map.

### Specimen collection and Virus isolation

Urine, throat swab and blood samples were collected from patients who had acute, febrile maculopapular rash from 24 provinces in China. All clinical samples were collected within five days of rash onset and transported in accordance with standard protocols [20,21]. Isolation of MeV was performed using the Vero/hSLAM cell line and the cells were harvested when the cytopathic effect (CPE) was visible over at least 50-75% of the cell layer [6,22].

### RNA Extraction and RT-PCR

RNA was extracted from 250 μl of infected cell lysate using a Trizol reagent, following the manufacturer's instructions. For all virus isolates, RT-PCR amplification was performed using previously described primers to amplify a 600bp fragment in the N gene which included the 450bp fragment recommended for genotyping [13]. PCR products were purified using the QIAquick Gel Extraction kit (QIAGEN).

### Sequence analysis

Sequences of the PCR products were derived by automated sequencing and the BigDye terminator v3.0 chemistry according to the manufacturer's protocol in both sense and antisense strands by an automated ABI PRISM™ 3100 DNA Sequencer (Perkin Elmer). Sequence proof reading and editing was conducted with Sequencer™ (Gene Codes Corporation). Sequence data were analyzed using version 7.0 of Bioedit and phylogenetic analyses were performed using Bioedit and Mega4 [23]. The robustness of the groupings was assessed using bootstrap resampling of 500 replicates and the trees were visualized with Mega programs. Nucleotide sequence data from 313 representative strains were deposited in GenBank under accession numbers: GU237175-GU237481

## List of Abbreviations

NNDRS: National Notifiable Diseases Reporting System; MeV: Measles virus; RT-PCR: reverse transcriptase polymerase chain reaction; CPE: cytopathic effect; N: Nucleoprotein; WHO: World Health Organization; WPR: Western Pacific Region;

## Competing interests

The authors declare that they have no competing interests.

## Authors' contributions

YXJ carried out most of the studies and drafted the manuscript. WBX designed the study and organized the coordination. YXJ, STX, YZ, ZZ and NYM performed parts of the studies and sequence analysis. XHJ, JHZ, PSL, CYW, YL, HYZ, collected specimens and performed virus isolation, viral identification. PAR and DAF provided consultation and editing of the manuscript. All authors read and approved the final manuscript.
